# CANDLE syndrome: A rare case report documented for the first time in the Middle East

**DOI:** 10.1097/MD.0000000000043124

**Published:** 2025-07-11

**Authors:** Hind Alhiraki, Mohammad Hamdi, Hossam Alhiraki, Ismail Alhiraki

**Affiliations:** a Children’s University Hospital, Faculty of Medicine, Damascus University, Damascus, Syria; b Department of Otolaryngology—Head and Neck Surgery, Al-Mouwasat University Hospital, Faculty of Medicine, Damascus University, Damascus, Syria; c Faculty of Medicine, Aleppo University, Aleppo, Syria.

**Keywords:** CANDLE syndrome, genetic test, skin lesions

## Abstract

**Rationale:**

CANDLE syndrome (chronic atypical neutrophilic dermatosis with lipodystrophy and elevated temperature) is an autoinflammatory disorder characterized by recurrent fever, skin lesions, and other symptoms caused by a mutation in the *PSMB8* gene.

**Patient concerns:**

This case report aims to describe the clinical features of a 3-year-old male patient with this syndrome. The patient, of Syrian origin, presented with recurrent fever and widespread skin lesions since the age of 7 months. There was a family history of similar skin lesions. On examination, erythematous eruptions and generalized lymphadenopathy were noted.

**Diagnoses:**

Genetic studies confirmed a homozygous nonsense mutation in PSMB8, a diagnostic of CANDLE syndrome. The patient showed symptomatic improvement with oral prednisolone.

**Interventions:**

The mutation associated with CANDLE syndrome is in PSMB8 (proteasome subunit β type 8), activated by interferon γ, and produces cytokines.

**Outcomes:**

This case is significant as it is the first reported CANDLE syndrome in Syria and the Middle East.

**Lessons:**

We highlight the variability in symptoms and responses to treatment and emphasize the noticeable improvement observed following treatment with corticosteroids alone.

## 1. Introduction

CANDLE syndrome (chronic atypical neutrophilic dermatosis with lipodystrophy and elevated temperature) is a rare genetic autoinflammatory disease caused by dysfunction of the proteasome-immunoproteasome complex.^[1]^ Autoinflammatory diseases and other related disorders have expanded.^[2]^ This syndrome was known as the NAKAJO-Nishimura syndrome and was first described by Nakajo in 1939 in Japan.^[3]^ The gene mutation associated with CANDLE syndrome is proteasome subunit β type 8 (PSMB8).^[4]^ In the early years of a child’s life, it is distinguished by recurrent fever, skin lesions, arthralgia, lipodystrophy, high levels of acute-phase proteins, and hypochromic or normocytic anemia.^[5]^ The treatment options for CANDLE syndrome include Janus kinase-signal transducer and activator of transcription 1/2 inhibitors and others, with noticeable improvement in some patients.^[6]^ Furthermore, there is no specific treatment for this syndrome, but there are drugs that may give some symptomatic improvement, such as oral corticosteroids, methotrexate, and nonsteroidal anti-inflammatory drugs. Systemic corticosteroids can be used in acute attacks.^[1]^ We report the case to be the first one in the Middle East, especially in Syria, where the patient was treated exclusively with corticosteroids. Our patient has symptoms, laboratory findings, and genetic studies that confirm the diagnosis of CANDLE syndrome.

## 2. Case presentation

**Figure 1.**
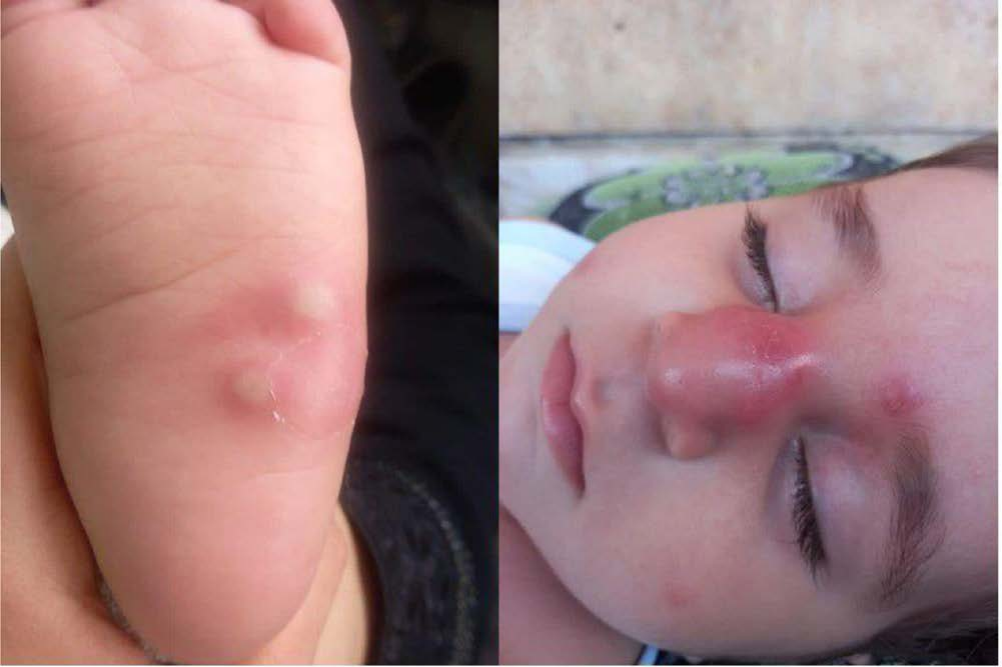
Two images of our patient showing lesions in different locations of the erythematous rash and nodules on the extremities.

**Figure 2.**
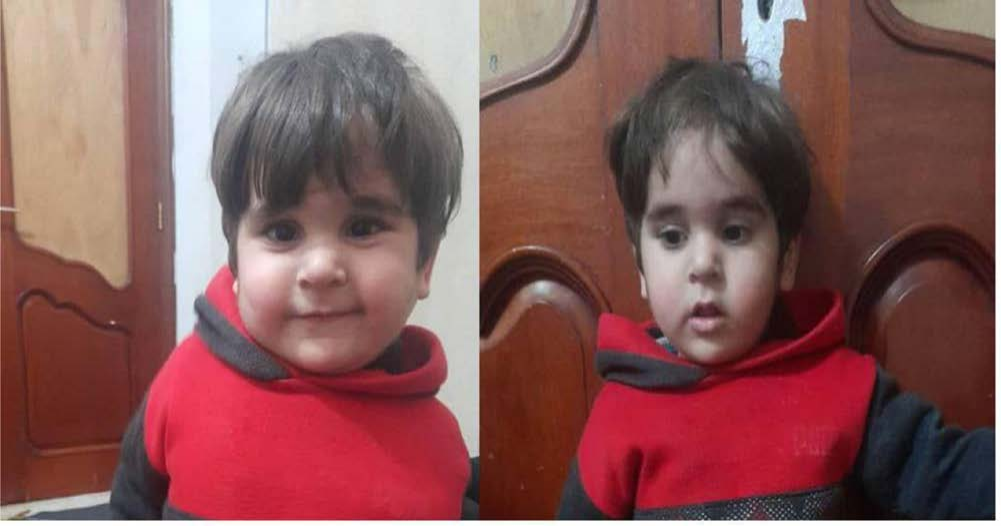
The same patient after receiving a course of oral prednisolone with symptomatic improvement.

A 3-year-old male presented to the children’s clinic with recurrent fever and widespread skin lesions since the age of 7 months. There is a family history of skin lesions. These symptoms have been recurring monthly. On physical examination, there are erythematous eruptions (Fig. 1) and generalized lymphadenopathy without hepatosplenomegaly or arthralgia. All other examinations were normal. He had been admitted to different hospitals where the laboratory tests revealed microcytic anemia, neutrophilic leukocytosis, low immunoglobulin levels, and high erythrocyte sedimentation rate and C-reactive protein levels, while serum alanine aminotransferase (ALT)/aspartate aminotransferase was normal. So, he was initially diagnosed with an immune deficiency and treated with antibiotics and intravenous immunoglobuline without any improvement. Subsequently, more specific tests were conducted, including a dihydrorhodamine flow cytometric test that showed chronic granulomatous disease. Quantitative lymphocyte subsets and immunoglobulins (CD3-CD4-CD8-CD16-CD19-CD56) were approximately normal. A genetic study showed a homozygous nonsense mutation in PSMB8 associated with CANDLE syndrome. Brain MRI and abdominal ultrasonography results were normal. Currently, we put the patient on oral prednisolone with symptomatic improvement and continued monitoring (Fig. 2).

## 3. Discussion

We document the first case of CANDLE syndrome in Syria and the Middle East. CANDLE syndrome is one of the autoinflammatory syndromes, as we mentioned, which has a wide spectrum of symptoms and clinical features, including recurrent fever, hypochromic or normocytic anemia, skin hyperpigmentation, and alopecia areata. The mutation associated with CANDLE syndrome is in PSMB8 (proteasome subunit β type 8), which is activated by interferon γ and produces cytokines. Thus, PSMB8 is related to several diseases, including autoimmune diseases, viral infections, and malignant tumors.^[7]^ In the study of Abhimanyu et al,^[8]^ the patient was 35 years old and was a normal infant, but during his childhood, there was a regression in his motor skills. Our patient was 3 years old, and the symptoms started at the age of 7 months, but without motor regression. CANDLE syndrome has a broad spectrum of symptoms. In the study of Mckenna et al,^[9]^ their patient had periodontal manifestations such as bone loss in maxillary and mandibular anterior teeth and horizontal bone loss in posterior teeth. Our patient has not manifested any of these symptoms. In the Yamazaki Nakashimada et al study, their patient had skin lesions, recurrent fever, and lymphadenopathy, and our patient has similar symptoms. However, their patient manifested hepatosplenomegaly and special facial features,^[10]^ which were absent in our patient. Regarding Asif Ali et al’s laboratory findings, they found that microcytic anemia, high erythrocyte sedimentation rate and C-reactive protein levels, elevated serum ALT, normal quantitative immunoglobulins, and lymphocyte subset (CD19-CD3-CD4-CD8-CD56). Genetic studies did not show a PSMB8 mutation, and they diagnosed their patient based on history and investigations.^[11]^ Our patient has laboratory findings similar to theirs, except for a normal level of serum ALT, and genetic studies showed a PSMB8 mutation. In CANDLE syndrome, there is activation of interferons by the Janus kinase-signal transducer and activator of transcription signaling pathway. Because of this, we can use JAK inhibitors in the treatment as suggested by Boyadzhiev et al^[12]^ in their study. In the previous study, they administered several therapies. They began with methylprednisolone, hydroxychloroquine, and methotrexate without any improvement. Then, they started with JAK inhibitors according to the NIH protocol. After 7 months of the treatment, they noticed significant improvement. We treated our patient with prednisolone and observed objective improvement. In conclusion, we have placed our patient under frequent monitoring to keep the symptoms under control and prevent any further progression.

## 4. Conclusion

This case report documents the first instance of CANDLE syndrome in Syria and the Middle East, highlighting the clinical features, diagnosis, and treatment of a 3-year-old male patient. The patient presented with recurrent fever and skin lesions, confirmed by a PSMB8 mutation. Symptomatic improvement was observed with oral prednisolone. This case underscores the importance of recognizing CANDLE syndrome in similar clinical scenarios. The treatment was limited to corticosteroids without using JAK inhibitors, and the therapy proved effective. Ongoing monitoring is essential for managing symptoms and preventing disease progression.

## 5. Limitations

The rarity of this disease, being the first reported case in the Middle East, along with the high cost of genetic testing, posed a significant challenge to diagnosis. Moreover, the high price and unavailability of JAK inhibitors limited our treatment plan to corticosteroids with regular follow-up.

## Author contributions

**Writing – original draft:** Hind Alhiraki, Mohammad Hamdi, Hossam Alhiraki.

**Supervision:** Ismail Alhiraki.

## References

[1] Torrelo A. CANDLE syndrome as a paradigm of proteasome-related autoinflammation. Front Immunol. 2017;8:927. 28848544 10.3389/fimmu.2017.00927 PMC5552674

[2] Figueras-Nart I Mascaró JM Jr Solanich X Hernández-Rodríguez J. Dermatologic and dermatopathologic features of monogenic autoinflammatory diseases. Front Immunol. 2019;10:2448. 31736939 10.3389/fimmu.2019.02448 PMC6828938

[3] Roberts T Stephen L Scott C. CANDLE syndrome: orodfacial manifestations and dental implications. Head Face Med. 2015;11:38. 26711936 10.1186/s13005-015-0095-4 PMC4693439

[4] Torrelo A Colmenero I Requena L. Histologic and immunohistochemical features of the skin lesions in CANDLE syndrome. Am J Dermatopathol. 2015;37:517–22. 26091509 10.1097/DAD.0000000000000340 PMC4476069

[5] Messia V Pardeo M Nicolai R Bracaglia C De Benedetti F Insalaco A. P02-016—a novel PSMB8 mutation causing candle syndrome. Pediatr Rheumatol Online J. 2013;11:A123–A123.

[6] Yu Q Mehta A Zou J. Human induced pluripotent stem cells generated from chronic atypical neutrophilic dermatosis with lipodystrophy and elevated temperature (CANDLE) syndrome patients with a homozygous mutation in the PSMB8 gene (NIHTVBi016-A, NIHTVBi017-A, NIHTVBi018-A). Stem Cell Res. 2022;62:102820. 35660921 10.1016/j.scr.2022.102820 PMC9514390

[7] Gomes AV. Genetics of proteasome diseases. Scientifica (Cairo). 2013;2013:637629. 24490108 10.1155/2013/637629 PMC3892944

[8] Garg A Hernandez MD Sousa AB. An autosomal recessive syndrome of joint contractures, muscular atrophy, microcytic anemia, and panniculitis-associated lipodystrophy. J Clin Endocrinol Metab. 2010;95:E58–63. 20534754 10.1210/jc.2010-0488 PMC2936059

[9] McKenna GJ Ziada HM. Periodontal manifestations of chronic atypical neutrophilic dermatosis with lipodystrophy and elevated temperature (CANDLE) syndrome in an 11-year-old patient. Clin Adv Periodontics. 2015;5:153–8. 32689728 10.1902/cap.2013.130071

[10] Yamazaki-Nakashimada MA Santos-Chávez EE de Jesus AA. Systemic autoimmunity in a patient with CANDLE syndrome. J Investig Allergol Clin Immunol. 2019;29:75–6. 10.18176/jiaci.0338 PMC8411438 30785112

[11] Ali M Islam M Rahman S. CANDLE syndrome: case report of a rare type of auto-inflammatory disease. Bangladesh J Child Health. 2021;44:174–7.

[12] Boyadzhiev M Marinov L Boyadzhiev V Iotova V Aksentijevich I Hambleton S. Disease course and treatment effects of a JAK inhibitor in a patient with CANDLE syndrome. Pediatr Rheumatol Online J. 2019;17:19. 31046790 10.1186/s12969-019-0322-9 PMC6498627

